# Interpersonal relationships of people living with HIV in sustained viral suppression with the multidisciplinary health team

**DOI:** 10.1590/0034-7167-2024-0490

**Published:** 2025-10-03

**Authors:** Thamyris Lucimar Pastorini Gonçalves, Maria Clara de Freitas, Mara Solange Gomes Dellaroza, Andressa Midori Sakai, Juliana Helena Montezeli, Gilselena Kerbauy

**Affiliations:** I Universidade Estadual de Londrina. Londrina, Paraná, Brazil

**Keywords:** Interpersonal Relations, Professional-Patient Relations, HIV Infections, Patient Care Team, Sustained Virologic Response., Relaciones Interpersonales, Relaciones Profesional-Paciente, Infecciones por VIH, Grupo de Atención al Paciente, Respuesta Virológica Sostenida.

## Abstract

**Objectives:**

to understand the discourse of people living with HIV in sustained viral suppression about their perceptions of interpersonal relationships with the multidisciplinary team.

**Methods:**

a qualitative study, carried out in two specialized healthcare services, which included 15 patients diagnosed with HIV in regular treatment since the beginning of antiretroviral therapy. Collection took place in 2022, from audio-recorded interviews. They were analyzed using the Discourse of the Collective Subject technique, with the support of IRaMuTeQ^®^.

**Results:**

speeches were prepared based on five classes. Some are: team role in linking patients to specialized services; holistic approach appreciation; patients’ reception, expectations and perspectives in relation to healthcare professionals; and concerns and challenges during treatment.

**Final Considerations:**

the results highlighted the importance of professionals for patients’ therapeutic success, especially when there is a good interpersonal relationship associated with the holistic approach, in addition to medicalization.

## RESUMEN

**Objetivos:**

comprender el discurso de personas viviendo con VIH en supresión viral sostenida respecto a sus percepciones de las relaciones interpersonales con el equipo multidisciplinario.

**Métodos:**

estudio cualitativo, realizado en dos servicios de salud especializados, que incluyó 15 pacientes con diagnóstico de VIH en tratamiento regular desde el inicio de la terapia antiretroviral. La recopilación se realizó en 2022, a partir de entrevistas grabadas en audio. Se analizaron utilizando la técnica de Discurso del Sujeto Colectivo, con el apoyo del software IRaMuTeQ^®^.

**Resultados:**

los discursos fueron elaborados con base en cinco clases. Algunos de ellos son: el papel del equipo en la vinculación de los pacientes con servicios especializados; valorando el enfoque holístico; Recepción, expectativas y perspectivas de los pacientes en relación con los profesionales de la salud; deseos y desafíos durante el tratamiento.

**Consideraciones Finales:**

los resultados resaltaron la importancia de los profesionales para el éxito terapéutico de los pacientes, especialmente cuando existe una buena relación interpersonal asociada al enfoque holístico, además de la medicalización.

## INTRODUCTION

Healthcare occurs through encounters among people; therefore, it is a social practice that requires interpersonal relationships to be well-woven so that the therapies employed can be effective. This applies to the most diverse care practices, and is especially important when it comes to individuals with chronic health conditions, such as people living with HIV/aids (PLWHA) and healthcare professionals in specialized services^(1)^.

In the universe of interpersonal relationships, communication undoubtedly emerges as a central tool, and this is an important skill that must be developed by healthcare professionals. Furthermore, the effective communication process among individuals in a society is an important tool for problem-solving and for developing good relationships between communities, teams and/or workers^(2)^.

In the context of care, it is essential that this process is effective in building interpersonal relationships between professionals and users. This attribute allows for open dialogue to obtain information that helps to resolve users’ needs, in addition to contributing to humanized attention and comprehensive care, from the prevention of injuries and illnesses to rehabilitation and/or treatment^(2)^.

In the health area, specifically in multidisciplinary work, a productive communicative performance is necessary to provide refined care, linked not only to the disease, but also to social issues, which can generate distress, anguish, social exclusion of the person being assisted and doubts regarding the finitude of life^(3)^. Moreover, weakness in communication among healthcare teams, as well as problems in interpersonal relationships during the provision of care and guidance to patients, can result in difficulties in coexistence and constant conflicts, which directly interfere with the recovery and treatment of individuals^(2)^.

Special emphasis should be given to people who require comprehensive and longitudinal care within the Brazilian Health System (In Portuguese, *Sistema Único de Saúde* - SUS), as in the case of PLWHA, whose clinical follow-up goes beyond the moment of diagnosis, requiring continuous monitoring. This population needs therapeutic continuity based on a care network that advocates not only timely diagnosis, but also linkage, retention and adherence to treatment, to achieve viral suppression and consequent success in antiretroviral therapy (ART), as proposed by the “continuous care cascade” for PLWHA, established globally and by the Ministry of Health (MoH)^(4)^.

There has been a significant increase in cases of HIV infection in the general population, and global data indicate that there are approximately 39 million people living with HIV worldwide; of these, 29.8 million were using ART in 2022^(5)^. In Brazil, there is an average annual increase of 36.4 reported cases of aids^(6)^.

In this context, the need for the creation and expansion of Specialized Assistance Services (SASs) in the outpatient setting emerged, institutions where it is urgent to strengthen bonds and interpersonal relationships between the multidisciplinary team and PLWHA. This is an important strategy for promoting adherence to treatment, based on the premise of regular appointments, regular use of antiretrovirals (ARVs), with the aim of suppressing the viral load (VL) from the beginning of treatment, carrying out periodic tests, in addition to preserving self-care and monitoring social issues that influence the appropriate use of medications^(7)^.

To structure this study, a literature review was carried out in the PubMed and CINAHL databases, observing that there is a large theoretical contribution regarding the difficulties faced by patients in using medication. However, little is said about the influence of interpersonal relationships with the multidisciplinary team on adherence to treatment and consequent therapeutic success.

Therefore, this research contributes to scientific literature by presenting the positive points that helped patients to achieve success in treatment, serving as a stimulus for the creation of public policies, assistance in the process of organizing care in specialized healthcare services, strengthening patients’ support network, and continuing education for healthcare professionals, especially with regard to the importance of well-established interpersonal relationships during treatment.

This study is justified by the need to understand how interpersonal relationships between PLWHA and the multidisciplinary health team are structured and established. It is known that patients’ bond with the team is essential for good adherence to treatment and adequate clinical management, as revealed by a study^(8)^ that addresses issues related to the ongoing care of these patients after HIV diagnosis. Moreover, monitoring, through a good team-patient relationship, becomes essential for assessing the health condition of these people and defining interventions that may be necessary throughout their lives^(8)^.

Therefore, given the ideas presented so far, the following guiding question was drawn up to conduct the present study: what is the discourse of PLWHA in sustained viral suppression about their interpersonal relationships with the multidisciplinary health team?

## OBJECTIVES

To understand the discourse of PLWHA in sustained viral suppression regarding their perceptions of interpersonal relationships with the multidisciplinary team.

## METHODS

### Ethical aspects

This research responds to one of the objectives of a project called “*Viva PositHIVo: promoção da qualidade de vida de pessoas que vivem com HIV*”, linked to the *Universidade Estadual de Londrina* and approved by the Research Ethics Committee. Participants were given explanations about the research objective, clarification of doubts, and reading and signing of the Informed Consent Form.

### Theoretical-methodological framework

This study is based on Serge Moscovici’s Theory of Social Representations (TSR), which takes into account the understanding of the world and the way people perceive and construct their realities. This theory allows the translation of perceptions about individuals’ individual and/or group experiences and which, in a certain way, represent a collective with respect to a given phenomenon, individual and/or object^(9)^.

### Study design

This is a qualitative study, organized according to the criteria recommended by the COnsolidated criteria for REporting Qualitative research^(10)^.

### Study setting and data source

The study was conducted in two SASs for the treatment of PLWHA, located in northern Paraná, Brazil. The services offer multidisciplinary care composed of infectious disease specialists, nurses, nursing technicians, social workers, psychologists and pharmacists.

Participants were selected by convenience among patients undergoing regular HIV treatment and in viral suppression since the start of ART who were present at the aforementioned services for consultations and/or test collections.

Individuals over 18 years of age (regardless of sex and education level) were included, those who had sustained viral suppression since the start of ART and who had been followed up at the SAS for at least one year. Those whose history of examinations attached to the medical record was incomplete were excluded.

### Data collection and organization

The data collection period took place between June and September 2022, through semi-structured interviews, which were conducted privately by the main researcher, with audio recording, and lasted 15 to 25 minutes. All information obtained was subject to confidentiality, with interviewees informed about anonymity and the use of a voice recorder.

To obtain the data, a semi-structured instrument divided into two stages was used: the first was carried out for participant sociodemographic characteristics and data from medical records to monitor the tests; and the second part consisted of three open-ended questions: 1) Tell me about your relationship with the health team from the diagnosis to the present; 2) For you, what is the role of the team in this process of reaching and sustained viral suppression?; 3) In your opinion, how should a healthcare professional relate to PLWHA?

### Data analysis

Data analysis was performed using the Discourse of the Collective Subject (DCS)^(11)^ and was performed by transcribing all interviews in full, listening to the audio and typing them literally into Microsoft Word^®^ for subsequent elaboration of the text *corpus*, which was processed using the *software Interface de R pour les Analyses Multidimensionnelles de Textes et de Questionnaires* (IRaMuTeQ^®^). This is a statistical program based on the R language that enables different types of analysis of content and textual elements. The statements were subjected to the semantic approximation process, in which the most relevant aspects of each question were organized and distributed in an understandable manner, resulting in graphical representations of the words most frequently uttered by participants^(12)^.

First, key expressions were identified, formulating the text *corpus*, according to procedures defined by IRaMuTeQ^®^. Then, the central ideas (CIs) (meaning of statements) were defined, as well as possible anchors (participants’ beliefs and/or values), based on the software analysis, and finally, the DSCs were constructed, written in a summarized manner, in the first person singular, with the purpose of representing collective thinking, aggregating in a single discourse the collective ways of thinking of the group, clarifying the collective opinions^(11)^.

To visualize the analysis of results generated by the software, the Descending Hierarchical Classification (DHC) graphical representation was chosen, which shows on a Cartesian plane the different words and variables associated with each of the classes generated. The text *corpus* inserted for this purpose must have a 70% utilization of text segments (TSs), a limit established by the literature to make the analysis more reliable. This type of interface makes it possible to visualize, in the original *corpus*, the TSs associated with each class and, thus, identify the context of the significant words for a more qualitative data analysis^(13)^.

## RESULTS

The study sample consisted of 15 PLWHA in sustained viral suppression. Males predominated, with 11 participants; participants’ ages ranged from 25 to 70 years; and the average treatment time for the group was seven years.

The key expressions entered into the software generated 84 TSs by IRaMuTeQ^®^, of which 63 were classified, resulting in a 75% utilization of the *corpus*. TSs are small parts of the text (text *corpus*), generally composed of three lines; after analysis, the software divides the *corpus* texts into TS to facilitate the categorization and meaning of entered data^(14)^.

It should also be noted that the TSs not classified by IRaMuTeQ^®^ were also made available, allowing for a detailed analysis of all content. The main researcher was able to verify that the excluded TSs did not alter the result, which favored the analysis reliability generated by the software.

**Figure 1:**
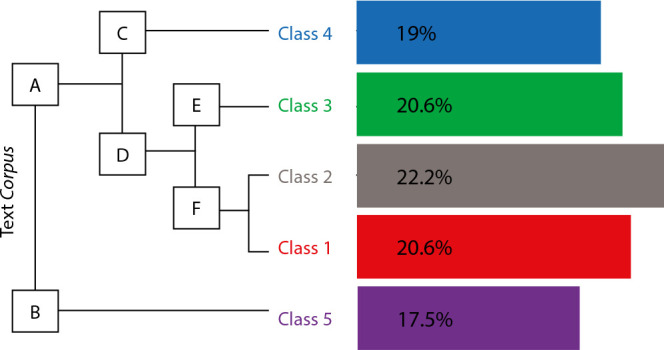
Class dendrogram graphical representation

A total of 2,933 occurrences (words, forms or vocabulary) emerged, with 556 lemmas (word standardization), 424 active forms (verbs, nouns and adjectives), 125 supplementary forms (articles, pronouns and adverbs) and 285 words with a single occurrence (called hapax). The analyzed content was categorized into five classes (class 1, with 13 TSs (20.63%); class 2, with 14 TSs (22.22%); class 3, with 13 TSs (20.63%); class 4, with 12 TSs (19.05%); and class 5, with 11 TSs (17.46%)), as shown in Figure 1.

The dendrogram represents the partition and size of each class in the text *corpus*, and the order of each class varies according to the repetition and connection among TSs. It is possible to observe in Figure 1 that the main *corpus* initially originated two *subcorpora* (A and B). *Subcorpus* A gave rise to branches C and D, with branch C promoting class 4, while D was subdivided into two other branches, E and F, which originated classes 3, 2 and 1, respectively. *Subcorpus* B, in turn, gave rise to class 5.

**Figure 2:**
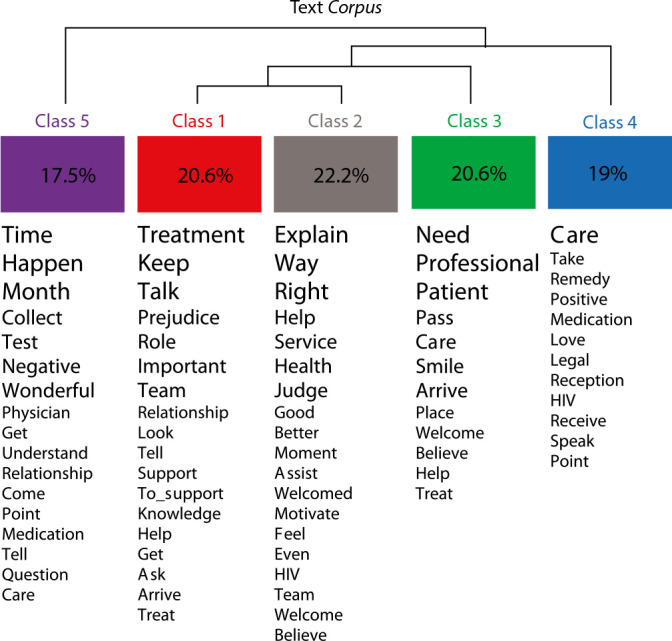
Descending Hierarchical Classification regarding the perception of people living with HIV/AIDS regarding their interpersonal relationships with the multidisciplinary team

From this, IRaMuTeQ^®^ generated the DHC of words that were most connected to each other (similar vocabularies) and appeared most frequently in the text *corpus*, making it possible to interpret the classes mentioned above, as shown in Figure 2.

Subsequently, the CIs were constructed based on the interpretation of the TSs of the five classes promoted by the software, in order to subsequently develop the DSCs. Chart 1 presents the CIs and DSCs of each class.

**Chart 1:** Composition of central ideas according to the classes developed and their speeches

Classs	Central ideas	Discourse of the Collective Subject
**Class 1**	The role of the multidisciplinary team in achieving and sustaining viral suppression, service linkage and the importance of professional discretion.	*It was great when I arrived here* (SAS). *I was well received, professionals were not prejudiced, I did not feel coerced and they supported me. I spoke to the psychologist, did the necessary tests, everything was planned so that I could continue with the treatment. The nurse who welcomed me played a very important role; he encouraged me with positive words. The conversation with the physician and the guidance from the team were important for me to understand how the treatment works. In addition, the discretion of everyone on the team helped me to continue coming here, because I know that there is a lot of ignorance regarding the knowledge that involves HIV, which is why I chose not to share my diagnosis. The team is always available, they never let me run out of medication and I feel at home here. During my treatment process to achieve viral suppression, the team always had a human approach. Their preparation for the circumstances helped me a lot. From the moment I was welcomed, I always had continuous assistance, which was essential in my journey. The professionals are here to support me not only in the area of ​​knowledge, but also in terms of friendship.*
**Class 2**	Health education as a strength for regular treatment.	*From the first moment I was approached, the service was excellent, everything was explained in a didactic manner. The professionals spoke in a simple way, without using difficult words, and the information was clear, both for those who understand HIV and those who don’t, and it was conveyed in a way that I understood. So, I think that’s the main point, because this initial welcome is what will motivate the person to continue treatment, and they really bring us closer together. When I was diagnosed at another service, it was difficult because they didn’t explain much to me, but when I got here, the technician who treated me calmed me down. It was a long process, but she was responsible for the first insight that this wasn’t the end and I felt well guided. I found a great sense of security here in the service. From the beginning, I always felt good, they always asked what they could do to help me, I never heard a joke.*
**Class 3**	Empathy as the main focus during care and assistance to PLWHA.	*The patient comes here with a broken mind, depressed, thinking about suicide. At first, for some, it is very difficult, so the professional needs to be willing to help and really make a difference, because they will see people coming in feeling bad and after a while, that same person will be wonderful. The professional needs to be empathetic, and put themselves in the shoes of the person who is sick, they need to know the patient’s history, be patient, charismatic and polite; this instills confidence. They helped me a lot with the emotional part, but I needed to focus, because there is no point in them helping if we do not recognize the support. It is important for the professional to always talk, ask if everything is okay, if they need help from any other area. If it is a physician, ask if they are interested in seeing a psychologist, a psychiatrist, if they want any other referral, because many people do not have family support. Therefore, the team needs to follow this line of care and treat people equally, regardless of their social status.*
**Class 4**	Valuing the holistic approach in the ongoing care of PLWHA.	*During my journey to achieve viral suppression, healthcare professionals fought alongside me, they always asked if I was taking my medication correctly. I feel important and remembered. I feel like they care about my life and my health. I think it’s great because this isn’t just a place where patients come to get medication. They want to take care of us and want everyone to have a happy ending. I’ve never wanted to stop coming here, I’ve always felt excited about coming because I feel welcomed; that makes me come back again and again and never stop, because I’ve received positive things that motivate me not to stop, that’s what makes the difference. It’s the smile, it’s the love. It’s the charisma that drives us. It’s a transmission of peace and joy in the soul.*
**Class 5**	Anxieties and challenges during the treatment of PLWHA.	*I think the issue of tests could be improved, because we are made up of ups and downs, and our viral load is only collected once a year. I think there should be more viral load collections. I believe that immunity can decrease in the meantime; I am more concerned about this part. One negative point is the pharmacy staff. When I come to pick up the medication, they sometimes don’t deliver a larger quantity; I have to come here every month. I also worry sometimes about, for example, if I can’t make it on the day of my appointment with the physician who is treating me and I can’t reschedule, because this also depends on whether I have some work commitment or unforeseen circumstances and I can’t make it, but most of the time, they understand. The physician is very careful about the medication part, when requesting tests, to know what is going on; he always answers the questions I bring. So, I think this is the most important thing, for us to understand what is really going on. Regarding the service, the only thing I can complain about is the lack of structural resources. I believe they would do even better if they had better working conditions, a more suitable space; I see a lack of a certain structure.*

## DISCUSSION

During the analysis of the TSs of each class for the preparation of CIs and subsequent DSC, it was possible to understand the different interfaces of PLWHA’s perceptions about their interpersonal relationships with the multidisciplinary health team, from the first contact in the discovery of diagnosis to the connection with the SAS. Based on speeches, it can be seen that trust in healthcare professionals and regularity in treatment, for most of patients interviewed, were well-established issues right from the first appointment, which can contribute to regularity in treatment.

The discourse related to class 1 mainly describes what contributed to the success of interviewees’ treatment and the consequent achievement and maintenance of undetectable VL; the adequate support that these people received was associated with the improvement in quality of life they had throughout treatment. This is in line with related literature, which identified that patients who received greater assistance and had well-established interpersonal relationships with healthcare professionals achieved more favorable results in treatment with regard to effective recovery after the start of therapy^(15)^. This finding reinforces the importance of qualified assistance based on humanized healthcare, with effective communication and free from prejudice and stigma.

Additionally, the importance of trust in the interpersonal relationship between patient and professional is highlighted, a fundamental feeling during consultations, as it favors adherence to the prescribed treatment and acceptance of health condition^(15)^. The social representation of the group studied supports this statement by revealing patients’ concern in maintaining discretion and confidentiality regarding diagnosis by healthcare professionals to favor regularity in treatment, demonstrating that it is a relevant and considerable factor in clinical monitoring.

Another aspect that proved to be essential in the collective discourse was in relation to patients’ understanding of the condition of HIV infection, both for those who already know the subject and for those who do not understand it, highlighting the need for a comprehensible approach that clarifies and transmits the knowledge necessary for effective health education. This point will be incorporated into the conception of TSR, which seeks to promote the reformulation of an unfamiliar concept into new knowledge that is useful and familiar to the general public^(16)^. Furthermore, users’ distance from the healthcare service may be associated with a lack of knowledge about issues inherent to HIV^(17)^.

In this context, it is worth highlighting the important role of health education, especially in situations where there is a need for personalized and educational care due to stigmas inherent to the infection. Although information is currently being passed on quickly, there is still an urgent need for educational technologies that increasingly promote care and self-care for the target audience, such as recreational activities that facilitate visualization of the HIV infection process, the importance and function of ARVs, among others^(18)^. To achieve this, it is necessary to seek innovative alternatives and proposals together with the multidisciplinary team, with the aim of involving knowledge and techniques from different areas.

The findings evidenced in the DSC of class 3 shed light on the fact that healthcare professionals’ empathy proved to be one of the great pillars for regularity in patient treatment. Respondents highlighted that empathy, ethical stance and emotional support, associated with adequate knowledge, are fundamental for quality of care in daily services. From this perspective, this set of characteristics is related to clinical management aimed at attentively listening to the demands presented by the group living with HIV and that promotes a relationship based on respect^(19)^.

The scope of the use of continuing education in specialized services as a powerful foundation in the outpatient routine is discussed, and it is questioned how professionals are being prepared to work in this type of care, since the study shows that such professionals are sought out daily to clarify doubts and are seen as a reference by users. In this context, ongoing educational processes are shown to be an intrinsic tool for the success of the work process of such healthcare professionals for the correct clinical-care management of PLWHA^(19)^.

Still in the same class, the group’s collective awareness of the importance of self-care is highlighted and that, in addition to the multidisciplinary team support, there is a need to understand the real health condition in order to achieve and sustain viral suppression.

A study on health literacy (HL) among PLWHA, which is the ability to read, understand, recognize and use health information, demonstrated the need to stimulate and develop patients’ personal skills to generate positive repercussions on continuity of care and adherence to ART. Although interventions to seek better HL rates do not always contribute significantly to removing people from unfavorable social conditions, they can reduce the impacts between social determination and health inequality in relation to living with HIV, since the level of education and income can interfere with the self-care of these people^(20)^.

Based on the TSR premise that each subject is primarily responsible for constructing their reality, based on the sociocultural context in which they are inserted, HL can be constituted as a tool for the implementation of care, becoming essential for the creation of public policies that promote knowledge and self-care of PLWHA^(20,21)^.

During the patient engagement process, it is important to include social determinants for an effective holistic approach, since, as highlighted in class 4, this is an important factor to be analyzed, especially with people undergoing treatment for chronic conditions. When HIV is diagnosed in the SAS, it becomes essential that professionals deal with the social conditions of each patient, going beyond the biomedical model, which focuses only on user medicalization^(22)^.

Therefore, multifactorial and interdisciplinary support is strategic not only in overcoming physical challenges, but also in aspects related to emotional problems involving HIV, in order to contribute to care directed at these people’s mental health. Furthermore, it is known that intersectorality and the union of different professionals increase the chance of ensuring a broad and integrated vision, offering more complete care in the healthcare process for PLWHA^(22)^.

In class 5, the DSC reveals that there are convergences regarding patients’ concerns about the frequency of VL collection, understanding of health status, and the importance of professionals’ flexibility in relation to the logistical obstacles that users face when going to the SAS. On the other hand, negative experiences with the pharmacy sector and specific concerns regarding rescheduling appointments emerge. This analysis highlights the complexity of social representations, reflecting the different perspectives and challenges through individuals’ experiences in relation to healthcare services.

Furthermore, the social representation of the group reveals the perception of the need for greater investment in the service’s structural resources. It is believed that it is important to consider an appropriate physical space not only for individual consultations, but also for activities that allow the team to interact with patients, relating the physical structure to the needs of projects involving different activities. These aspects can favor the comprehensiveness of healthcare and the promotion of patients’ and professionals’ well-being. Therefore, it is essential to highlight the fragility of the current organizational work process and its causes so that strategies compatible with the service’s reality can emerge^(23)^.

Regarding the frequency of medication withdrawal, specialized services have a standard established by the MoH. According to the protocol, ARVs should be withdrawn every 30, 60 and 90 days, depending on the clinical conditions and phases of treatment. It is recommended that the frequency be defined through biannual consultations or at the discretion of the physician in charge^(24)^.

According to the MoH policies^(24)^, VL test collection should be monitored according to PLWHA’s clinical situation. In the case of people in viral suppression with clinical and immunological stability, the frequency of requesting the test is every six months, always with the main objective of confirming the viral situation and patient adherence. For laboratory monitoring of the CD4+ lymphocyte test (LT-CD4+) and for people with results above 500 cells per mm^3^ of blood in two consecutive tests, at least six months apart, it is recommended that it not be requested as a daily routine.

In this context, it is observed that there are still weaknesses in the care and management of problems experienced daily by PLWHA. This is reflected in the organizational culture exercised in the aforementioned service, with the aim of strengthening user bonding, highlighting the need for a flexible work method that considers the uniqueness of each individual, in order to personalize care for the target audience. In this logic, it is essential to identify the strategies recommended by the MoH and used by the SAS to bond patients and, thus, promote the qualification of managers and healthcare professionals who work in this area of care^(25)^.

From this perspective, it is important that the team has autonomy in managing care, with the aim of individualizing conduct and avoiding rigid routines. But it also clarifies to the user the limits of possible flexibility, since some changes may be impossible due to structural issues in the system.

### Study limitations

This research had as a limitation the fact that it was not possible to assess patients’ socioeconomic aspects, in order to relate the data to the discourses elaborated, which would allow a broader view of the lived reality of users. However, it still proves relevant to support advances in the search for strategies that promote multidimensional, subjective and individual care for PLWHA.

### Contributions to health

The study emphasizes the aspects that were essential for adherence to treatment by people living with HIV, in order to clarify gaps in care and reveal the social representation of the perspectives, strengths and obstacles inherent to the study population. Thus, the understanding of the positive aspects experienced by patients during treatment was expanded, in addition to expanding knowledge about practices of healthcare services that helped in adherence to ART.

These identified dimensions may assist in the development of health interventions based on the psychosocial dynamics of PLWHA and, thus, contribute to the improvement and strengthening of specialized healthcare services and support networks available in the SUS as well as to the realignment of therapeutic plans for those people who do not adhere to treatment adequately.

## FINAL CONSIDERATIONS

The objectives of this study were achieved as the speeches pointed to the main aspects that contributed to regular treatment, revealing the primordial role that healthcare professionals play in the therapeutic success of patients with regard to good interpersonal relationships. However, some challenges that still need to be overcome were also highlighted, mainly regarding the organizational work process of healthcare services, such as weaknesses in work routines and, in some cases, the lack of flexibility in decisions made by some SAS sectors.

It was possible to observe that patients value a holistic approach and that this contributes to their connection to healthcare services and consequent success in treatment. Thus, the need for studies and interventions that encompass aspects of the daily life of PLWHA that are intrinsic to treatment and that go beyond medicalization is reinforced.

It is believed that the data presented here can serve as a contribution to critical reflections by healthcare professionals regarding the type of assistance that has been offered to the target audience, in addition to favoring the creation of public policies that address the complexity of the topic discussed.

## Data Availability

The research data are available within the article.
