# Supplementary material for: Interpersonal relationships of people living with HIV in sustained viral suppression with the multidisciplinary health team
Source: Rev Bras Enferm. 2025 Oct 3;78(Suppl 2):e20240490. doi: 10.1590/0034-7167-2024-0490 (PMC12500069; doi:10.1590/0034-7167-2024-0490)
Supplement: Translation

# Translation: Relações interpessoais da pessoa vivendo com HIV em supressão viral sustentada com a equipe multidisciplinar de saúde

**DOI:** 10.1590/0034-7167-2024-0490pt

**Authors:** Thamyris Lucimar Pastorini Gonçalves, Maria Clara de Freitas, Mara Solange Gomes Dellaroza, Andressa Midori Sakai, Juliana Helena Montezeli, Gilselena Kerbauy

**Affiliations:** I Universidade Estadual de Londrina. Londrina, Paraná, Brasil

**Keywords:** Relações Interpessoais, Relações Profissional-Paciente, Infecções por HIV, Equipe de Assistência ao Paciente, Supressão Viral Sustentada.

## Abstract

**Objetivos:**

apreender o discurso da pessoa vivendo com HIV em supressão viral sustentada acerca de suas percepções sobre as relações interpessoais com a equipe multidisciplinar.

**Métodos:**

estudo qualitativo, realizado em dois serviços especializados de saúde, que incluiu 15 pacientes com diagnóstico de HIV em tratamento regular desde o início da terapia antirretroviral. A coleta ocorreu em 2022, a partir de entrevistas audiogravadas. Foram analisadas pela técnica do Discurso do Sujeito Coletivo, com apoio do *software IRaMuTeQ^®^*.

**Resultados:**

os discursos foram elaborados a partir de cinco classes. São eles: o papel da equipe na vinculação dos pacientes ao serviço especializado; valorização da abordagem holística; acolhimento, expectativas e perspectivas dos pacientes em relação aos profissionais de saúde; anseios e desafios durante o tratamento.

**Considerações Finais:**

os resultados apontaram a importância dos profissionais para o sucesso terapêutico dos pacientes, principalmente quando há um bom relacionamento interpessoal associado à abordagem holística, além da medicalização.

## INTRODUÇÃO

O cuidado em saúde ocorre por meio do encontro entre pessoas; logo, trata-se de uma prática social que requer que as relações interpessoais sejam bem tecidas para que haja efetivação das terapêuticas empregadas. Isso se aplica às mais diversas práticas de cuidado, e tem especial importância quando se trata de indivíduos portadores de condições crônicas de saúde, como a pessoa que vive com HIV/aids (PVHA) e os profissionais de saúde dos serviços especializados^(1)^.

No universo das relações interpessoais, indubitavelmente, a comunicação emerge como ferramenta central, sendo esta uma importante competência que deve ser desenvolvida pelos profissionais de saúde. Ademais, o processo de comunicação efetivo entre os indivíduos de uma sociedade é importante ferramenta para a resolução de problemas e para o desenvolvimento de um bom relacionamento entre comunidades, equipes e/ou trabalhadores^(2)^.

No âmbito do cuidado, é primordial que haja a efetividade desse processo para a construção de relações interpessoais entre profissionais e usuários. Tal atributo permite o diálogo aberto para obtenção de informações que auxiliam na resolutividade das necessidades dos usuários, além de contribuir com uma atenção humanizada e um cuidado integral, desde a prevenção de agravos e doenças até reabilitação e/ou tratamento^(2)^.

Na área da saúde, especificamente no trabalho multidisciplinar, é necessário um desempenho comunicativo profícuo para proporcionar um atendimento refinado, atrelado não somente à doença, mas também às questões sociais, que podem gerar sofrimentos, angústias, exclusão social da pessoa assistida e dúvidas em relação à finitude da vida^(3)^. Em complemento, a fragilidade na comunicação das equipes de saúde, bem como problemas nas relações interpessoais durante a realização dos cuidados e orientações aos pacientes, pode resultar em dificuldades de convivência e conflitos constantes, os quais interferem diretamente na recuperação e tratamento dos indivíduos^(2)^.

Especial destaque deve ser dado às pessoas que demandam cuidado integral e longitudinal no âmbito do Sistema Único de Saúde (SUS), como no caso da PVHA, cujo seguimento clínico vai além do momento do diagnóstico, requerendo um acompanhamento contínuo. O público em questão necessita de uma continuidade terapêutica alicerçada em uma rede de atenção que preconiza não somente o diagnóstico no momento oportuno, mas também a vinculação, retenção e adesão ao tratamento, para alcance da supressão viral e consequente êxito na terapia antirretroviral (TARV), como proposto pela “cascata de cuidado contínuo” da PVHA, instituída globalmente e pelo Ministério da Saúde (MS)^(4)^.

Há importante crescimento nos casos de infecção pelo HIV na população em geral, e dados globais indicam que há cerca de 39 milhões de pessoas vivendo com HIV no mundo; destas, 29,8 milhões estavam em uso da TARV no ano de 2022^(5)^. No Brasil, há em média um crescimento anual de 36,4 casos de AIDS notificados^(6)^.

Nesse contexto, emergiu a necessidade da criação e expansão de Serviços de Assistência Especializada (SAEs) no âmbito ambulatorial, instituições onde urge a primordialidade do fortalecimento de vínculos e relações interpessoais entre equipe multidisciplinar e PVHA. Trata-se de uma importante estratégia para a promoção da adesão ao tratamento, partindo da premissa de assiduidade nas consultas, uso regular dos antirretrovirais (ARVs), com o intuito de suprimir a carga viral (CV) desde o início do tratamento, realização de exames periódicos, além da preservação do autocuidado e acompanhamento de questões sociais que influenciam o uso adequado das medicações^(7)^.

Para estruturação do presente estudo, realizou-se revisão de literatura nas bases de dados PubMed e CINAHL, observando-se que há um grande aporte teórico em relação às dificuldades enfrentadas pelos pacientes no uso da medicação. Entretanto, pouco se fala acerca da influência das relações interpessoais com a equipe multidisciplinar na adesão ao tratamento e consequente êxito terapêutico.

Portanto, esta pesquisa contribui com a literatura científica ao apresentar os pontos positivos que ajudaram os pacientes a obter êxito no tratamento, servindo de fomento para criação de políticas públicas, auxílio no processo de organização do cuidado nos serviços especializados de saúde, fortalecimento da rede de apoio dos pacientes, e educação permanente para os profissionais de saúde, principalmente no que diz respeito à importância de relações interpessoais bem estabelecidas durante o tratamento.

Este estudo é justificado pela necessidade de se compreender como as relações interpessoais entre a PVHA e a equipe multidisciplinar de saúde são estruturadas e estabelecidas. Sabe-se que a vinculação do paciente com a equipe é essencial para uma boa adesão ao tratamento e manejo clínico adequado, como revela estudo^(8)^ que aborda questões relacionadas ao cuidado contínuo desses pacientes após o diagnóstico do HIV. Além disso, o acompanhamento, por meio de uma boa relação equipe-paciente, torna-se primordial para avaliação da condição de saúde dessas pessoas e definição de intervenções que forem necessárias ao longo da vida^(8)^.

Destarte, diante das ideias até aqui tecidas, traçou-se a seguinte questão norteadora para conduzir o presente estudo: qual o discurso da PVHA em supressão viral sustentada sobre suas relações interpessoais com a equipe multidisciplinar de saúde?

## OBJETIVOS

Apreender o discurso da PVHA em supressão viral sustentada acerca de suas percepções sobre as relações interpessoais com a equipe multidisciplinar.

## MÉTODOS

### Aspectos éticos

Esta pesquisa responde a um dos objetivos do projeto “Viva PositHIVo: promoção da qualidade de vida de pessoas que vivem com HIV”, vinculado à Universidade Estadual de Londrina e aprovado pelo Comitê de Ética em Pesquisa. Aos participantes, foram fornecidas explicações acerca do objetivo da investigação, esclarecimento das dúvidas, bem como leitura e assinatura do Termo de Consentimento Livre e Esclarecido.

### Referencial teórico-metodológico

Trata-se de estudo alicerçado na Teoria das Representações Sociais (TRS) de Serge Moscovici, que leva em consideração a compreensão do mundo e o modo como as pessoas percebem e constroem suas realidades. Essa teoria permite a tradução de percepções sobre as vivências individuais e/ou grupais dos indivíduos e que, de certo modo, representam uma coletividade a respeito de um determinado fenômeno, indivíduo e/ou objeto^(9)^.

### Desenho do estudo

Trata-se de estudo de abordagem qualitativa, cuja organização seguiu os critérios preconizados pelo *COnsolidated criteria for REporting Qualitative research* ^(10)^.

### Cenário do estudo e fonte de dados

O estudo foi desenvolvido em dois SAEs para o tratamento de PVHA, que ficam localizados no norte do estado do Paraná, Brasil. Os serviços oferecem um cuidado multidisciplinar composto por médicos infectologistas, enfermeiros, técnicos de enfermagem, assistentes sociais, psicólogos e farmacêuticos.

A seleção dos participantes ocorreu por conveniência entre os pacientes em tratamento regular para HIV e em supressão viral desde o início da TARV que estavam presentes nos referidos serviços para realização de consultas e/ou coletas de exames.

Incluíram-se indivíduos maiores de 18 anos (independentemente do sexo e escolaridade), os que possuíam supressão viral sustentada desde o início da TARV e que estavam em acompanhamento no SAE por pelo menos um ano. Excluíram-se aqueles cujo histórico de exames anexados no prontuário estava incompleto.

### Coleta e organização dos dados

O período de coleta de dados aconteceu entre os meses de junho e setembro de 2022, por meio de entrevistas semiestruturadas, que foram conduzidas de maneira privativa pela pesquisadora principal, com gravação de áudio, e tiveram duração de 15 a 25 minutos. Todas as informações obtidas foram sujeitas ao sigilo, sendo os entrevistados informados sobre o anonimato e o uso do gravador de voz.

Para obtenção dos dados, foi utilizado instrumento semiestruturado dividido em duas etapas: a primeira foi realizada para caracterização sociodemográfica dos participantes e dados do prontuário para acompanhamento dos exames; e a segunda parte foi constituída por três perguntas abertas: 1) Conte-me sobre a sua relação com a equipe de saúde desde o diagnóstico até o momento; 2) Para você, qual o papel da equipe nesse processo de alcance e supressão viral sustentada?; 3) Na sua opinião, como um profissional de saúde deve se relacionar com a PVHA?

### Análise dos dados

A análise dos dados foi feita pelo Discurso do Sujeito Coletivo (DSC)^(11)^, e ocorreu a partir da transcrição na íntegra de todas as entrevistas, com escuta dos áudios e digitação literal no programa *Microsoft Word* ^®^ para posterior elaboração do *corpus* textual, o qual foi tratado com apoio do *software Interface de R pour les Analyses Multidimensionnelles de Textes et de Questionnaires* (IRaMuTeQ^®^). Este é um programa estatístico baseado na linguagem *R,* que viabiliza diferentes tipos de análises de conteúdos e elementos textuais. As falas foram submetidas ao processo de aproximação semântica, em que os aspectos mais relevantes de cada pergunta foram organizados e distribuídos de maneira compreensível, resultando em representações gráficas das palavras mais proferidas pelos participantes^(12)^.

Primeiramente, foram identificadas as expressões-chave, formulando-se o *corpus* textual, conforme procedimentos definidos pelo *IRaMuTeQ^®^.* Então, procedeu-se à definição das ideias centrais (ICs) (sentido dos depoimentos), eventuais ancoragens (crenças e/ou valores dos participantes), a partir da análise do *software*, e, por fim, a construção dos DSCs redigidos, de maneira resumida, na primeira pessoa do singular, com a finalidade de representar o pensamento da coletividade, agregando em um discurso único modos coletivos de pensar do grupo, clarificando as opiniões coletivas^(11)^.

Para visualização da análise dos resultados gerados pelo *software*, foi escolhida a representação gráfica da Classificação Hierárquica Descendente (CHD), que evidencia em um plano cartesiano as diferentes palavras e variáveis associadas a cada uma das classes geradas. O *corpus* textual inserido para este fim precisa possuir um aproveitamento de 70% dos segmentos de texto (STs), limite estabelecido pela literatura para tornar a análise mais fidedigna. Esse tipo de interface possibilita visualizar, no *corpus* original, os STs associados a cada classe e, assim, identificar o contexto das palavras significativas para uma análise mais qualitativa dos dados^(13)^.

## RESULTADOS

A amostra do estudo foi composta por 15 PVHAs em supressão viral sustentada. Predominou o sexo masculino, com 11 participantes; a idade dos participantes variou entre 25 e 70 anos; e o tempo médio de tratamento do grupo foi de sete anos.

As expressões-chave inseridas no *software* geraram 84 STs pelo *IRaMuTeQ^®^*, dos quais 63 foram classificados, resultando em um aproveitamento de 75% do *corpus*. Os STs são pequenas partes do texto (*corpus* textual), geralmente compostos por três linhas; após a análise, o *software* divide os textos do *corpus* em ST para facilitar a categorização e os sentidos dos dados inseridos^(14)^.

Salienta-se ainda que os STs não classificados pelo *IRaMuTeQ^®^* também foram disponibilizados, permitindo a análise minuciosa de todo o conteúdo. A pesquisadora principal pôde verificar se os STs excluídos não alteraram o resultado, o que favoreceu a fidedignidade da análise gerada pelo *software.*

Emergiram 2.933 ocorrências (palavras, formas ou vocábulos), com 556 lemas (padronização das palavras), 424 formas ativas (verbos, substantivos e adjetivos), 125 formas suplementares (artigos, pronomes e advérbios) e 285 palavras com uma única ocorrência (denominadas de *hapax*). O conteúdo analisado foi categorizado em cinco classes (classe 1, com 13 STs (20,63%); classe 2, com 14 STs (22,22%); classe 3, com 13 STs (20,63%); classe 4, com 12 STs (19,05%); e classe 5, com 11 STs (17,46%)), conforme evidenciado na Figura 1.

**Figura 1:** Representação gráfica do dendrograma de classes

O dendrograma representa a partição e o tamanho de cada classe referente ao *corpus* textual, e a ordem de cada uma delas varia de acordo com a repetição e conexão entre os STs. É possível observar na Figura 1 que o *corpus* principal originou, inicialmente, dois *subcorpora* (A e B). O *subcorpus* A deu origem às ramificações C e D, sendo que a ramificação C promoveu a classe 4, enquanto a D se subdividiu em outras duas ramificações, a E e a F, que originaram as classes 3, 2 e 1, respectivamente. O *subcorpus* B, por sua vez, deu origem à classe 5.

A partir disso, o *IRaMuTeQ^®^* gerou a CHD das palavras que mais se conectaram entre si (vocabulários semelhantes) e apareceram com mais ocorrência no *corpus textual*, sendo possível a interpretação das classes citadas acima, como mostrado na Figura 2.

**Figura 2:** Classificação Hierárquica Descendente acerca da percepção da pessoa que vive com HIV/aids sobre suas relações interpessoais com a equipe multidisciplinar

Em seguida, construíram-se as ICs com base na interpretação dos STs das cinco classes promovidas pelo *software*, a fim de elaborar, na sequência, os DSCs. O Quadro 1 apresenta as ICs e os DSCs de cada classe.

**Quadro 1:** Composição das ideias centrais conforme as classes elaboradas e os seus discursos

Classes	Ideias centrais	Discurso do Sujeito Coletivo
**Classe 1**	O papel da equipe multidisciplinar no alcance e sustentação da supressão viral, vinculação do serviço e a importância da discrição dos profissionais.	*Foi muito bom quando eu cheguei aqui* (SAE)*. Fui bem recebido, os profissionais não tiveram preconceito, não me senti coagido e eles me apoiaram. Conversei com a psicóloga, fiz os exames necessários, foi tudo elaborado para que eu conseguisse manter o tratamento. O enfermeiro que me acolheu teve um papel importantíssimo; ele me estimulou com palavras positivas. A conversa com o médico e a orientação da equipe foram importantes para eu entender como funciona o tratamento. Além disso, a discrição de todos da equipe contribuiu para eu continuar vindo até aqui, pois eu sei que há muita ignorância em relação ao conhecimento das coisas que envolvem o HIV, por isso eu optei por não compartilhar meu diagnóstico. A equipe sempre está à disposição, nunca deixa faltar medicamentos e aqui eu me sinto em casa. Durante meu processo de tratamento para alcance da supressão viral, a equipe sempre teve um olhar humano. O preparo deles diante das circunstâncias me ajudou bastante. Desde o momento que eu fui acolhido, sempre tive uma assistência contínua, o que foi primordial em minha trajetória. Os profissionais estão aqui para apoiar não só na área do conhecimento, mas também em relação à amizade.*
**Classe 2**	Educação em saúde como fortaleza para um tratamento regular.	*Desde a primeira abordagem, o atendimento foi de excelência, tudo foi explicado de maneira didática. A forma simples de falar dos profissionais, sem palavras difíceis, a informação clara, tanto para quem entende de HIV como para quem não entende, foi passada de uma maneira que eu compreendi. Então, eu acho que esse é o ponto principal, pois essa primeira acolhida é o que vai motivar a pessoa a continuar o tratamento, e eles realmente trazem a gente para mais perto. Quando eu recebi o diagnóstico em outro serviço, foi difícil porque não me explicaram quase nada, mas quando cheguei aqui, a técnica que me atendeu me acalmou. Foi um processo demorado, mas ela foi a responsável pelo primeiro insight de que isso não era o fim e eu me senti bem orientado. Eu encontrei aqui uma segurança muito grande no serviço. Desde o início, eu sempre me senti bem, sempre perguntam o que podem fazer para me ajudar, nunca escutei uma piada.*
**Classe 3**	A empatia como foco principal durante os atendimentos e cuidado da PVHA.	*O paciente entra aqui com a mente abalada, em depressão, pensando em suicídio. No começo, para alguns, é muito difícil, então o profissional precisa estar disposto a ajudar e realmente fazer a diferença, porque ele vai ver pessoas chegando mal e depois de um tempo, essa mesma pessoa vai estar maravilhosa. O profissional precisa ser empático, e se colocar no lugar de quem fica doente, precisa saber do histórico do paciente, ter paciência, carisma e ser educado; isso passa confiança. Eles me ajudaram bastante na parte emocional, mas eu precisei ter foco, pois não adianta nada eles ajudarem e a gente não reconhecer o apoio. É importante o profissional sempre conversar, perguntar se está tudo bem, se precisa da ajuda de alguma outra área. Se for médico, perguntar se tem interesse em ir ao psicólogo, um psiquiatra, se quer algum outro encaminhamento, porque muitas pessoas não têm apoio familiar. Por isso, a equipe precisa seguir nessa linha de cuidado e tratar as pessoas de maneira igual, independentemente da condição social.*
**Classe 4**	Valorização da abordagem holística no cuidado contínuo da PVHA.	*Durante a minha caminhada para alcançar a supressão viral, os profissionais de saúde lutaram junto comigo, eles sempre perguntavam se eu estava tomando o remédio do jeito certo. Eu me sinto importante e lembrado. Eu sinto que eles cuidam da minha vida e da minha saúde. Eu acho legal porque aqui não é só um lugar onde os pacientes vêm buscar remédio. Eles querem cuidar e querem que todos tenham um final feliz. Eu nunca tive vontade de não vir aqui, sempre me senti entusiasmado em vir porque me sinto acolhido; isso faz com que eu volte sempre e não pare, porque recebi coisas positivas isso me motiva a não parar, isso é o diferencial. É o sorriso, é o amor. é o carisma que nos impulsiona. É uma transmissão de paz e de alegria na alma.*
**Classe 5**	Anseios e desafios durante o tratamento da PVHA.	*Acho que pode melhorar a questão dos exames, porque a gente é feito de altos e baixos, e nossa carga viral é colhida apenas uma vez por ano. Acho que deveria ter mais coletas de carga viral. Acredito que a imunidade pode cair nesse meio tempo; eu fico mais preocupado nessa parte. Um ponto negativo são as funcionárias da farmácia. Quando eu venho retirar os medicamentos, acontece de não entregarem uma quantidade maior, tem que vir todo mês aqui. A minha preocupação às vezes também é em relação, por exemplo, se eu não conseguir vir no dia da consulta com o médico que me acompanha e não ter como remarcar, porque isso também depende se eu tiver algum compromisso do trabalho, imprevistos e não for possível vir, mas na maioria das vezes, eles entendem. O médico tem bastante cuidado nessa parte da medicação na parte de solicitação de exames para saber o que está acontecendo; ele sempre responde as dúvidas que eu levo. Então, eu acho que isso é o mais importante, a gente entender o que realmente está acontecendo. Do serviço, o que eu posso reclamar é da falta de recurso estrutural. Eu acredito que eles fariam até melhor se tivessem mais condições de trabalho, um espaço mais adequado; eu vejo a escassez de uma certa estrutura.*

## DISCUSSÃO

Durante a análise dos STs de cada classe para elaboração das ICs e posterior DSC, foi possível compreender as diferentes interfaces das percepções da PVHA sobre as suas relações interpessoais com a equipe multidisciplinar de saúde, desde o primeiro contato na descoberta do diagnóstico até a vinculação com o SAE. Baseando-se nos discursos, pode-se perceber que a confiança no profissional de saúde e a regularidade no tratamento, para a maioria dos pacientes entrevistados, foram questões bem estabelecidas logo no primeiro atendimento, o que pode contribuir para regularidade no tratamento.

O discurso referente à classe 1 descreve principalmente o que contribuiu para êxito no tratamento dos entrevistados e consequente alcance e sustentação da CV indetectável; associou-se o adequado acolhimento que essas pessoas receberam à melhora da qualidade de vida que tiveram ao longo do tratamento. Isso vai ao encontro com a literatura correlata, a qual identificou-se que pacientes envoltos por uma maior assistência e relações interpessoais bem estabelecidas com os profissionais de saúde alcançaram resultados mais favoráveis no tratamento no que diz respeito a uma recuperação efetiva após o início da terapia^(15)^. Esse achado reforça a importância da assistência qualificada alicerçada na atenção à saúde humanizada, com comunicação eficaz e livre de preconceitos e estigmas.

Aditivamente, ressalta-se a importância da confiança no relacionamento interpessoal paciente-profissional, sentimento fundamental durante os atendimentos, pois favorece a adesão ao tratamento prescrito e aceitação da condição de saúde^(15)^. A representação social do grupo estudado corrobora essa afirmativa ao revelar a preocupação dos pacientes em manter a discrição e o sigilo sobre o diagnóstico, por parte dos profissionais de saúde, para favorecer a regularidade no tratamento, demonstrando ser um fator relevante e considerável no acompanhamento clínico.

Outro aspecto que se mostrou essencial no discurso coletivo foi em relação ao entendimento dos pacientes sobre a condição da infecção pelo HIV, tanto para quem já conhece o assunto quanto para quem não o compreende, despontando a necessidade de uma abordagem compreensível que esclareça e transmita o conhecimento necessário para uma efetiva educação em saúde. Esse apontamento vai se incorporar à concepção da TRS, que busca promover a reformulação de um conceito não familiar para um novo conhecimento útil e familiar ao grande público^(16)^. Ademais, o distanciamento do usuário com o serviço de saúde pode estar associado ao desconhecimento de assuntos inerentes ao HIV^(17)^.

É válido destacar, nesse contexto, o importante papel da educação em saúde, principalmente em situações em que há necessidade de um atendimento didático e particularizado, devido aos estigmas intrínsecos à infecção. Embora, atualmente, o repasse de informações ocorra de maneira rápida, ainda urge a necessidade de tecnologias educacionais que favoreçam cada vez mais a promoção do cuidado e autocuidado do público-alvo, como atividades lúdicas que facilitem a visualização do processo de infecção do HIV, importância e função dos ARVs, entre outros^(18)^. Para isso, é necessário buscar alternativas e propostas inovadoras juntamente com a equipe multidisciplinar, com o intuito de envolver saberes e técnicas de diferentes áreas.

Os achados evidenciados no DSC da classe 3 dão luz ao fato de que a empatia dos profissionais de saúde se mostrou como um dos grandes pilares para a regularidade no tratamento dos pacientes. Os inquiridos destacaram que a empatia, a postura ética e o apoio emocional, associados ao conhecimento adequado, são fundamentais para a qualidade do atendimento no cotidiano dos serviços. Nessa perspectiva, relaciona-se esse conjunto de características ao manejo clínico voltado a uma escuta atenta às demandas apresentadas pelo grupo que vive com HIV e que promova uma relação pautada no respeito^(19)^.

Discute-se, assim, a dimensão do uso da educação permanente nos serviços especializados como poderoso alicerce na rotina ambulatorial, e indaga-se de que maneira os profissionais estão sendo preparados para atuar nesse tipo de cuidado, pois o estudo mostra que tais profissionais são diariamente procurados para esclarecimento de dúvidas e vistos como referência pelos usuários. Nesse contexto, os processos educacionais contínuos se mostram como uma ferramenta intrínseca ao sucesso do processo de trabalhado de tais profissionais de saúde para o correto manejo clínico-assistencial da PVHA^(19)^.

Ainda na mesma classe, é evidenciada a consciência coletiva que o grupo tem da importância do autocuidado e de que, além do apoio da equipe multidisciplinar, há necessidade de compreender a real condição de saúde para o alcance e sustentação da supressão viral.

Estudo acerca do letramento em saúde (LS) da PVHA, que é a habilidade em ler, compreender, reconhecer e usar as informações de saúde, demonstrou a necessidade de estimular e desenvolver habilidades pessoais dos pacientes para gerar repercussões positivas na continuidade do cuidado e adesão à TARV. Embora as intervenções para busca de melhores índices de LS nem sempre contribuam significativamente para a retirada das pessoas de condições sociais desfavoráveis, elas podem diminuir os impactos entre a determinação social e a desigualdade em saúde no que se refere a viver com HIV, uma vez que o nível de formação e renda pode interferir no autocuidado dessas pessoas^(20)^.

Partindo da premissa da TRS de que cada sujeito é o principal responsável pela construção de sua realidade, a partir do contexto sociocultural que está inserido, o LS pode se constituir como ferramenta para efetivação do cuidado, tornando-se imprescindível para criação de políticas públicas que promovam o conhecimento e autocuidado da PVHA^(20,21)^.

Durante o processo de vinculação do paciente, é considerável incluir os determinantes sociais para uma abordagem holística efetiva, pois, como destaca-se na classe 4, este é um importante fator a ser analisado, sobretudo com pessoas em tratamento de condições crônicas. Quando há o diagnóstico de HIV no SAE, torna-se essencial que os profissionais lidem com as condições sociais de cada paciente, indo além do modelo biomédico, que foca apenas na medicalização do usuário^(22)^.

Portanto, o apoio multifatorial e interdisciplinar é estratégico não apenas na superação de desafios físicos, mas também de aspectos relacionados aos problemas emocionais que envolvem o HIV, a fim de contribuir com cuidados direcionados à saúde mental dessas pessoas. Ademais, sabe-se que a intersetorialidade e a união de diferentes profissionais aumentam a chance de garantir uma visão ampla e integrada, oferecendo cuidados mais completos no processo de atenção à saúde da PVHA^(22)^.

Na classe 5, o DSC revela que há convergências em relação à preocupação dos pacientes sobre a frequência na coleta de CV, à compreensão do estado de saúde e à importância da flexibilidade que os profissionais possuem em relação aos obstáculos logísticos que os usuários enfrentam para irem até o SAE. Por outro lado, surgem experiências negativas tidas com o setor de farmácia e nas inquietações específicas a respeito da remarcação de consultas. Essa análise evidencia a complexidade das representações sociais, refletindo as diferentes perspectivas e desafios mediante experiências dos indivíduos em relação aos serviços de saúde.

Além disso, a representação social do grupo revela a percepção da necessidade de maiores investimentos também em recursos estruturais do serviço. Acredita-se na importância de se pensar em um espaço físico apropriado não só para consultas individualizadas, mas também para atividades que proporcionem a interação da equipe com os pacientes, relacionando a estrutura física com as necessidades de projetos de diferentes atuações. Esses aspectos podem favorecer a integralidade da assistência à saúde e a promoção do bem-estar dos pacientes e profissionais. Assim, é primordial que seja sinalizada a fragilidade do atual processo organizacional de trabalho e suas causas para o surgimento de estratégias compatíveis com a realidade do serviço^(23)^.

No que diz respeito à periodicidade da retirada dos medicamentos, os serviços especializados possuem um padrão instituído pelo MS. De acordo com o protocolo, a retirada dos ARVs deve ser realizada entre 30, 60 e 90 dias, a depender das condições clínicas e fases do tratamento, recomendando-se que a frequência seja definida por meio das consultas semestrais ou a critério do médico responsável^(24)^.

De acordo com as políticas do MS^(24)^, a coleta de exame de CV deve ser monitorada mediante a situação clínica da PVHA. No caso de pessoas em supressão viral com estabilidade clínica e imunológica, a frequência de solicitação do exame é a cada seis meses, sempre com o objetivo principal de confirmar a situação viral e adesão do paciente. Para o monitoramento laboratorial do exame de linfócitos CD4+ (LT-CD4+) e das pessoas com resultado acima de 500 células por mm^3^ de sangue em dois exames consecutivos, com pelos menos seis meses de intervalo, preconiza-se que não seja solicitado como rotina diária.

Diante desse contexto, observa-se que ainda há fragilidades no cuidado e manejo de problemas vivenciados cotidianamente pelas PVHAs. Isso se reflete na cultura organizacional exercida no referido serviço, com o intuito de fortalecer a vinculação do usuário, destacando a necessidade de um método de trabalho flexibilizado que considere a singularidade de cada indivíduo, de modo a personalizar o atendimento do público-alvo. Nessa lógica, é imprescindível identificar as estratégias recomendadas pelo MS e utilizadas pelo SAE para vincular os pacientes e, assim, promover a qualificação de gestores e profissionais de saúde que atuam nessa área do cuidado^(25)^.

Nessa perspectiva, é importante que a equipe tenha autonomia de gestão do cuidado, com o objetivo de individualizar a conduta e evitar o engessamento de rotinas. Mas também esclarece ao usuário o limite da flexibilização possível, já que algumas mudanças podem ser impossíveis por questões estruturais do sistema.

### Limitações do estudo

Esta pesquisa trouxe como limitação o fato de não ter sido possível avaliar os aspectos socioeconômicos dos pacientes, a fim de relacionar os dados aos discursos elaborados, o que permitiria uma visão mais ampla sobre a realidade vivida dos usuários. Porém, ainda assim, mostra-se relevante para subsidiar avanços na busca por estratégias que promovam um cuidado multidimensional, subjetivo e individual à PVHA.

### Contribuições para a área da saúde

O estudo enfatiza os aspectos que foram primordiais para adesão ao tratamento das pessoas que vivem com HIV, de modo a clarificar as lacunas do cuidado e desvelar a representação social acerca das perspectivas, fortalezas e obstáculos inerentes ao público do estudo. Assim, expandiu-se a compreensão dos pontos positivos vivenciados pelos pacientes durante o tratamento, além de ampliar os conhecimentos sobre as práticas dos serviços de saúde que auxiliaram na adesão à TARV.

Essas dimensões identificadas poderão auxiliar na elaboração de intervenções de saúde pautadas na dinâmica psicossocial da PVHA e, assim, contribuir com o aperfeiçoamento e fortalecimento dos serviços especializados de saúde e das redes de apoio disponíveis no SUS, bem como para o realinhamento dos planos terapêuticos àquelas pessoas que não aderem ao tratamento de maneira adequada.

## CONSIDERAÇÕES FINAIS

Os objetivos do presente estudo foram alcançados à medida que os discursos apontaram para os principais aspectos que contribuíram para um tratamento regular, revelando a primordialidade do papel que os profissionais de saúde desempenham para o sucesso terapêutico dos pacientes no que diz respeito a um bom relacionamento interpessoal. Contudo, foram evidenciados também alguns desafios que ainda precisam ser superados, principalmente no tocante ao processo organizacional de trabalho dos serviços de saúde, como fragilidades nas rotinas de trabalho e, em alguns casos, a ausência da flexibilidade nas decisões tomadas por alguns setores do SAE.

Foi possível constatar que os pacientes valorizam uma abordagem holística e que isso contribui para vinculação dos mesmos aos serviços de saúde e consequente êxito no tratamento. Dessa maneira, reforça-se a necessidade de estudos e intervenções que englobem os aspectos do cotidiano da PVHA intrínsecos ao tratamento que vão além da medicalização.

Acredita-se que os dados aqui apresentados possam servir de contribuição para reflexões críticas dos profissionais de saúde a respeito do tipo de assistência que vem sendo oferecida ao público-alvo, além de favorecer a criação de políticas públicas que abordem a complexidade do tema discutido.

## Data Availability

Os dados da pesquisa estão disponíveis no corpo do artigo.
